# Differences in cardiac involvement between carriers of Duchenne and Becker muscular dystrophy - a cardiovascular magnetic resonance study

**DOI:** 10.1186/1532-429X-17-S1-Q90

**Published:** 2015-02-03

**Authors:** Anca R Florian, Anna Ludwig, Sabine Rösch, Udo Sechtem, Ali Yilmaz

**Affiliations:** 1 Cardiology, Uniklinikum Muenster, Muenster, Germany; 2 Cardiology, Robert Bosch Hospital, Stuttgart, Germany

## Background

Duchenne (DMD) and Becker (BMD) muscular dystrophies are X-linked recessive disorders associated with both skeletal myopathy and progressive cardiomyopathy in males. Although BMD patients present milder skeletal muscle involvement, they have been shown to present more advanced cardiomyopathy than DMD. Female DMD/BMD carriers are usually free of skeletal muscle symptoms but they may also develop cardiomyopathy.

The present study aimed at characterizing the presence and pattern of cardiac abnormalities in a group of genetically-proven MD-carriers by means of comprehensive CMR studies. Moreover, we wanted to test whether DMD carriers (DMDc) and BMD carriers (BMDc) are differently affected.

## Methods

Thirty-six (age 44±14yrs) female MD-carriers (20 DMDc and 16 BMDc) were prospectively enrolled. All patients underwent a multi-parametric CMR study comprising cine- and LGE-CMR (1.5-Tesla). A left ventricular (LV) LV ejection fraction (LV-EF) <55% and/or presence of LGE defined a pathologic CMR study.

## Results

In the total study group, mean LV-EF was 64±9%. Seventeen (47%) patients had at least one pathological CMR finding based on the CMR results: 6 (17%) patients demonstrated (at least) a reduced LV-EF and 16 (44%) patients showed (at least) presence of LGE. All LGE positive patients (N=16) showed non-ischemic LGE with subepicardial involvement of the LV lateral wall as most frequent pattern (13/16, 81%). Compared to BMDc, DMDc were significantly younger (40±11yrs vs. 50±16yrs, p=0.038) and presented significantly higher LV end-systolic volumes (53±18mL/m2 vs. 40±13mL/m2, p=0.025) in addition to lower LV-EF (62±8% vs. 68±9%, p=0.039). Among DMDc, 65% (N=13) showed presence of LGE compared to only 19% (N=3) in BMDc (p=0.008). Taken together, a pathologic CMR result was found in 65% (N=13) of DMDc compared to only 25% (N=4) in BMDc (p=0.023).

## Conclusions

Cardiac involvement is a frequent finding in female carriers of DMD, but rarely observed in carriers of BMD. Both, DMDc and BMDc with cardiac involvement demonstrate the same myocardial fibrosis pattern as their male counterparts with overt disease. Interestingly, in contrast to what is seen in male patients with DMD and BMD, female carriers of DMD present with a more advanced cardiomyopathy than carriers of BMD.

## Funding

None.

